# The Tricalbin-Family Endoplasmic Reticulum-Plasma Membrane Tethering Proteins Attenuate ROS-Involved Caspofungin Sensitivity in Candida albicans

**DOI:** 10.1128/spectrum.02079-22

**Published:** 2022-11-29

**Authors:** Li Yang, Hangqi Zhu, Mingchun Li, Qilin Yu

**Affiliations:** a Key Laboratory of Molecular Microbiology and Technology, Ministry of Education, Department of Microbiology, College of Life Sciences, Nankai University, Tianjin, People’s Republic of China; University of Iowa Hospitals and Clinics

**Keywords:** *Candida albicans*, reactive oxygen species, endoplasmic reticulum-plasma membrane contact, tricalbin, drug sensitivity, drug tolerance, fungal pathogen, oxidative stress

## Abstract

The endoplasmic reticulum-plasma membrane (ER-PM) contacts are one kind of important membrane contact structures in eukaryotic cells, which mediate material and message exchange between the ER and the PM. However, the specific types and functions of ER-PM tethering proteins are poorly understood in the human fungal pathogen Candida albicans. In this study, we observed that the two tricalbin-family proteins, i.e., Tcb1 and Tcb3, were colocalized with the ER-PM contacts in C. albicans. Deletion of the tricalbin-encoding genes *TCB1* and *TCB3* remarkably reduced ER-PM contacts, suggesting that tricalbins are ER-PM tethering proteins of C. albicans. Stress sensitivity assays showed that the *TCB*-deleted strains, including *tcb1*Δ/Δ, *tcb3*Δ/Δ, and *tcb1*Δ/Δ *tcb3*Δ/Δ, exhibited hypersensitivity to cell wall stress induced by caspofungin. Further investigation revealed that caspofungin induced drastic reactive oxygen species (ROS) accumulation in the mutants, which was attributed to enhanced oxidation of Ero1 in the ER lumen. Removal of intracellular ROS by the ROS scavenger vitamin C rescued the growth of the mutants under caspofungin treatment, indicating that Ero1 oxidation-related ROS accumulation was involved in caspofungin hypersensitivity of the mutants. Moreover, deletion of the *TCB* genes decreased secretion of extracellular aspartyl proteinases, reduced transport of the cell wall protein Hwp1 from the cytoplasm to the cell wall, and attenuated virulence of the fungal pathogen. This study sheds a light on the role of ER-PM tethering proteins in maintenance of cell wall integrity and virulence in fungal pathogens.

**IMPORTANCE** The endoplasmic reticulum-plasma membrane contacts are important membrane contact structures in eukaryotic cells, functioning in material and message exchange between the ER and the PM. We observed that the two tricalbin-family endoplasmic reticulum-plasma membrane contact proteins are required for tolerance to caspofungin-induced cell wall stress in the pathogenic fungus Candida albicans. The tricalbin mutants exhibited hypersensitivity to cell wall stress induced by caspofungin. Further investigation revealed that Ero1 oxidation-related reactive species oxygen accumulation was involved in caspofungin hypersensitivity of the tricalbin mutants. Moreover, loss of tricalbins reduced secretion of extracellular aspartyl proteinases, decreased transport of the cell wall proteins from the cytoplasm to the cell wall, and attenuated virulence of the fungal pathogen. This study uncovers the role of ER-PM tethering proteins in sustaining protein secretion, maintenance of cell wall integrity and virulence in fungal pathogens.

## INTRODUCTION

The endoplasmic reticulum-plasma membrane (ER-PM) contacts, one of the most important membrane contacts, are prevalent linkers between the ER and the PM (1). This type of contacts has diverse functions, such as lipid and ion transport, signal transduction, and regulation of enzyme activity (2–5). In Saccharomyces cerevisiae, ER-PM contacts are mediated by several kinds of ER-PM tethering proteins to form the linkers with 20 to 30 nm distance (6, 7), which are involved in calcium homeostasis regulation and lipid transport (3, 8, 9). Tricalbin-family proteins are conserved ER-PM tethering proteins that locate on the cortical ER (cER) adjacent to PM. These proteins play an important role in formation of ER-PM contacts. In S. cerevisiae cells, there are three tricalbins, i.e., Tcb1, Tcb2, and Tcb3. Deletion of the genes encoding tricalbins leads to abnormal cER morphology and reduced resistance to heat stress (10–15), indicating their role in stress response. However, their role in the pathogenic fungi remains to be investigated.

C. albicans is a clinically important opportunistic pathogenic fungus (16). It exists as a harmless commensal fungus in the oral or gastrointestinal tract of many healthy individuals (17). However, in immunocompromised patients, the fungus may cause superficial infections and even systemic infections in bloodstream and internal organs, which are frequently associated with high rates of mortality (18, 19). The cell wall on the surface of C. albicans cells directly interacts with the host and is closely related to the pathogenicity of C. albicans (20). It is involved in a series of virulence-related processes, such as biofilm formation, maintenance of osmotic pressure, adhesion, and morphogenesis on the host skin surface (21). The cell wall of C. albicans consists of polysaccharides (β-1,3-glucan, β-1,6-glucan, chitin, mannan) and cell wall proteins that are specific to this pathogen and therefore is an ideal target of antifungal drugs (18, 22). For example, caspofungin specifically inhibits the synthesis of β-1,3-glucan in the fungal cell wall and therefore inhibits fungal growth (23).

Cell wall components are synthesized in the ER and then transported to the cell wall by secretory vesicles via the classical secretory pathway (24). Owing to the critical role of the ER and the PM in maintenance of cell wall integrity (CWI), the fungal cells evolve complicated mechanisms to remodel the functions of the ER and the PM to attenuate cell wall stress. For instance, cell wall stress may promote protein expression and folding, which further activates unfolded protein response (UPR) and enhances the oxidation of Ero1 in the ER (25). No doubt, as the bridge between the ER and the PM, ER-PM contacts may be involved in cell wall stress response by regulating material transport. However, the function of ER-PM contacts in cell wall stress tolerance remains to be detailed in fungal pathogens.

This study aimed to investigate the role of the tricalbins in cell wall stress tolerance and virulence in C. albicans. Initially, we found that there are only two tricalbins in this pathogen, named Tcb1 and Tcb3, which are encoded by orf 19.1840 (i.e., *TCB1*) and orf 19.3003 (i.e., *TCB3*), respectively. The single mutant *tcb1*Δ/Δ and *tcb3*Δ/Δ, together with the double mutant *tcb1*Δ/Δ *tcb3*Δ/Δ, were then constructed using PCR-mediated homologous recombination. The role of Tcb1 and Tcb3 in formation of ER-PM contacts, cell wall stress tolerance, morphogenesis, and systemic infection was then investigated by using the mutants. Our finding revealed the novel role of ER-PM contacts in maintenance of cell wall integrity and pathogenicity in fungal pathogens.

## RESULTS

### Deletion of TCB genes attenuates ER-PM contacts in C. albicans.

**FIG 1:**
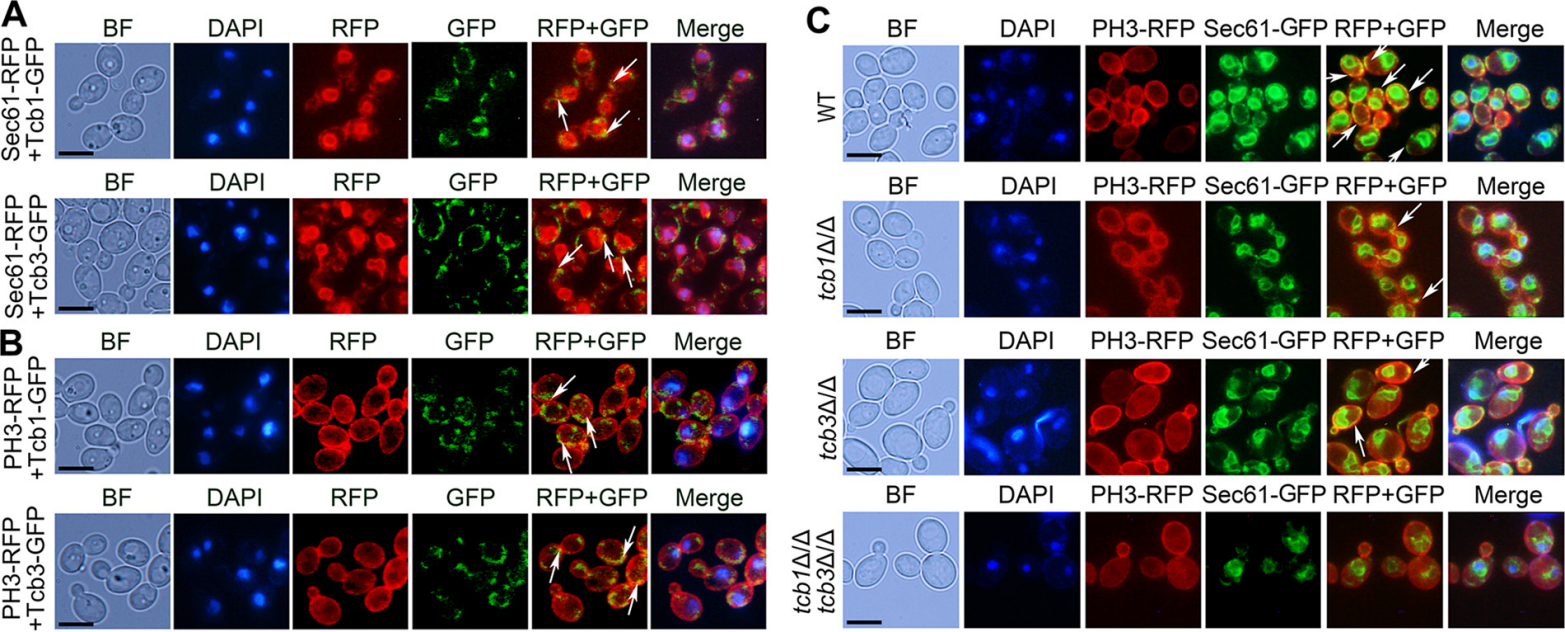
Localization of tricalbins and effect of their disruption on formation of ER-PM contacts in C. albicans. (A) Co-localization of Tcb1-GFP and the ER marker Sec61-RFP (up, indicated by the arrows) in the WT+Tcb1-GFP Sec61-RFP strain, and colocalization of Tcb3-GFP and Sec61-RFP (down, indicated by the arrows) in the WT+Tcb3-GFP Sec61-RFP strain. (B) Colocalization of Tcb1-GFP and the PM marker PH3-RFP (up, indicated by the arrows) in the WT+Tcb1-GFP PH3-RFP strain, and colocalization of Tcb3-GFP and PH3-RFP (down, indicated by the arrows) in the WT+Tcb3-GFP PH3-RFP strain. The cells were stained by DAPI to indicate the nucleus. Scale bar = 5 μm. (C) Colocalization of the ER marker Sec61-GFP and the PM marker PH3-RFP in WT, *tcb1*Δ/Δ, *tcb3*Δ/Δ and *tcb1*Δ/Δ *tcb3*Δ/Δ. The arrows indicate the representative ER-PM contacts. Scale bar = 5 μm. Note that WT possesses the highest levels of ER-PM contacts.

In S. cerevisiae, the ER-PM tethering proteins are located on the cortical ER (cER) adjacent to the PM (26). In order to determine whether tricalbins are ER-PM tethering proteins of C. albicans, the intracellular localization of tricalbins in C. albicans were first investigated by labeling the tricalbins with GFP. Fluorescence microscopy showed that the two tricalbins, i.e., Tcb1 and Tcb3, partially colocalized with the ER marker Sec61-RFP (Fig. 1A), and with the PM marker PH3-RFP (Fig. 1B), indicating that the two tricalbins were located on the cER adjacent to the PM.

To further explore the role of the tricalbins in formation of the ER-PM contacts, both Sec61 and PH3 were labeled by GFP and RFP in WT, *tcb1*Δ/Δ, *tcb3*Δ/Δ, *tcb1*Δ/Δ, *tcb3*Δ/Δ strains, respectively. Since Sec61-GFP and PH3-RFP show distribution of the ER and the PM, respectively, the sites with colocalization of Sec61-GFP and PH3-RFP indicate the ER-PM contacts. The distribution of ER-PM contacts in these strains was then observed by fluorescence microscopy. In the initial BWP17 (WT) strain, Sec61-GFP and PH3-RFP colocalizing sites were extremely rich, indicating the enrichment of ER-PM contacts. In contrast, the sites with colocalization of Sec61-GFP and PH3-RFP obviously decreased in *tcb1*Δ/Δ, *tcb3*Δ/Δ, *tcb1*Δ/Δ, *tcb3*Δ/Δ strains (Fig. 1C). Moreover, as revealed by statistical analysis, the WT cells had a much higher percentage of ER-PM contact-displaying cells than the mutant cells (92% versus 23% for *tcb1*Δ/Δ, 18% for *tcb3*Δ/Δ, and 3% for *tcb1*Δ/Δ *tcb3*Δ/Δ, Fig. S1), indicating that deletion of *TCB* impaired ER-PM contacts. Thus, the two tricalbins are ER-PM tethering proteins, and play an important role in formation of the ER-PM contacts in C. albicans.

### Deletion of *TCB* genes results in hypersensitivity to caspofungin.

**FIG 2:**
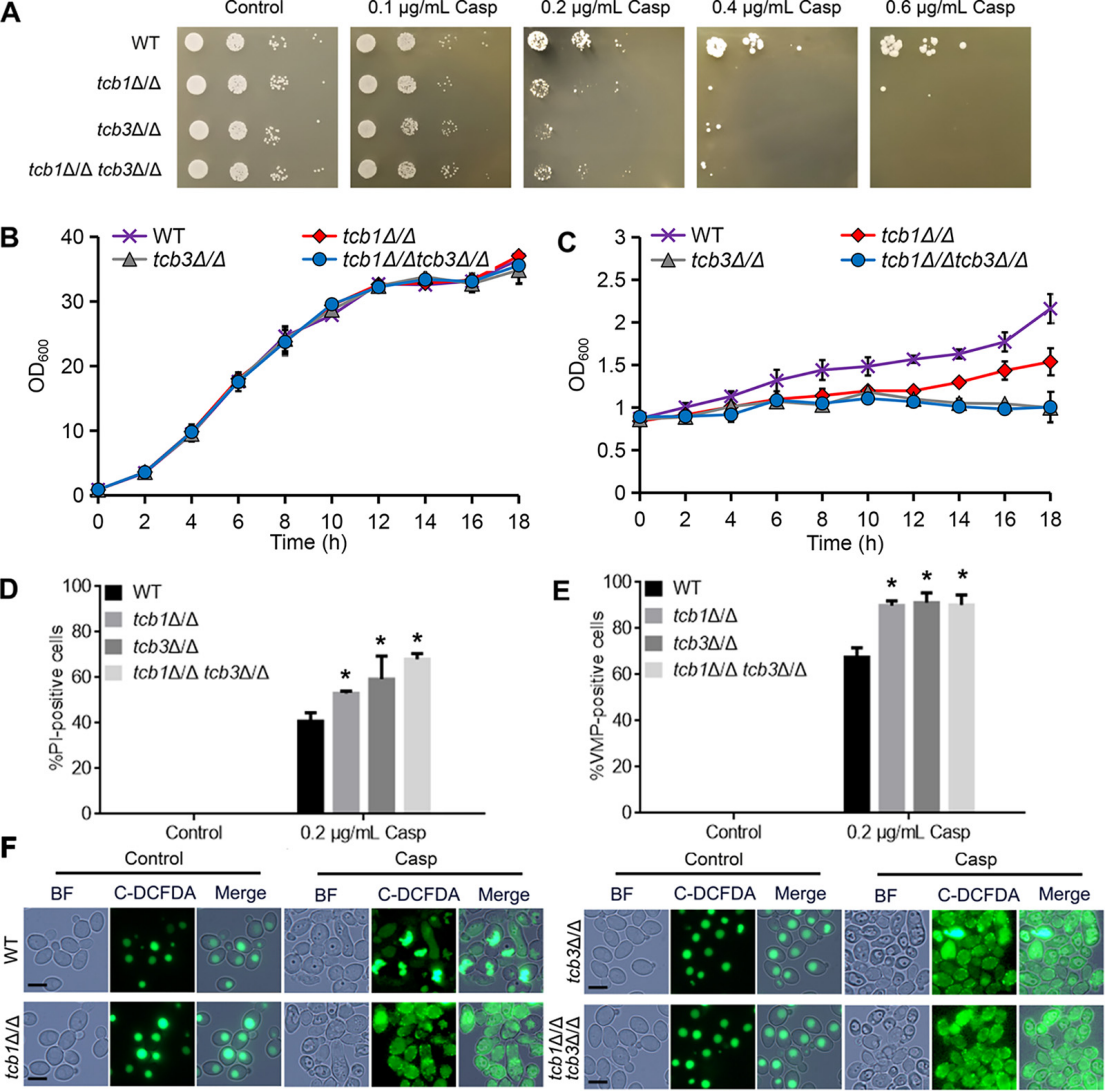
Effect of *TCB* deletion on growth under caspofungin (Casp) treatment. (A) Growth of the strains on solid YPD medium or the medium containing Casp at the indicated concentrations. The plates were incubated at 30°C and photographed after 2 days. (B) Growth curves of WT, *tcb1*Δ/Δ, *tcb3*Δ/Δ, and *tcb1*Δ/Δ *tcb3*Δ/Δ cultured in liquid YPD medium at 30°C and 160 rpm. (C) Growth curves of WT, *tcb1*Δ/Δ, *tcb3*Δ/Δ and *tcb1*Δ/Δ *tcb3*Δ/Δ in the normal liquid YPD medium containing 0.2 μg/mL Casp. (D) PM damage assays. The cells were cultured in YPD medium or the medium containing 0.2 μg/mL Casp for 2 h. The cells were collected and stained with PI for fluorescence microscopy. The percentage of PI-positive cells was calculated. At least 10 fields were observed. (E) Statistical analysis of VMP-positive cells. At least 10 fields were observed for analysis. (F) Fluorescence microscopy images of VMP-positive cells. Bar = 5 μm. The treated cells were stained by C-DCFDA and observed by fluorescence microscopy. *, significant difference between the mutants and WT, *P* < 0.05.

Cell wall is a very critical structure of fungi, playing an important role in many physiological processes, such as resistance to osmotic pressure, maintenance of mechanical strength, recognition of environmental signals, and interaction with the host tissues (21). The ER is the place in which many cell wall components are synthesized, modified, and released (25) and therefore is essential for maintenance of CWI. To investigate the link between tricalbins and CWI, we first evaluated the sensitivity of the *TCB* mutants to caspofungin, a clinical drug inhibiting synthesis of cell wall β-1, 3-glucan. As shown in Fig. 2A, the *TCB* mutants, including *tcb1*Δ/Δ, *tcb3*Δ/Δ, *tcb1*Δ/Δ, *tcb3*Δ/Δ, exhibited hypersensitivity to caspofungin at a concentration at 0.2 μg/mL and above, indicating that tricalbins contributed to the tolerance of C. albicans to this cell wall-perturbing drug. In order to confirm the role of tricalbins in caspofungin tolerance, the growth rate of the mutants was detected in liquid YPD medium containing caspofungin or not. In the caspofungin-free YPD medium, there was no significant difference in the growth rate between WT and the *TCB* deletion mutants (Fig. 2B). However, in the medium containing 0.2 μg/mL caspofungin, the three *TCB* mutants exhibited a much lower growth rate than WT. Especially, the growth of *tcb3*Δ/Δ and *tcb1*Δ/Δ *tcb3*Δ/Δ was almost completely inhibited by caspofungin (Fig. 2C). These results indicated that tricalbins are required for caspofungin tolerance of this fungal pathogen.

The inhibitory effect of antifungal drugs on fungal growth is closely associated with enhancement of PM damage and vacuolar membrane permeability (VMP) (27, 28). PI staining was first performed to evaluate PM damage. In the normal YPD medium, the four tested strains had no PI-positive cells, indicating these strains had intact PM. However, under caspofungin treatment at the concentration of 0.2 μg/mL, all tested cells had a great percentage of PI-positive cells, and the three *TCB* mutants exhibited higher levels of PI-positive cells than WT (55 to 64% versus 40%, Fig. 2D). Similarly, as revealed by C-DCFDA staining and fluorescence microscopy, the three *TCB* mutants had a higher percentage of VMP-positive cells with whole-cell distribution of C-DCFDA than WT (>90% versus <70%, Fig. 2E and F). In addition, caspofungin treatment at the concentration of 0.6 μg/mL, which drastically inhibited the growth of WT (Fig. 2A), also resulted in high levels of both PI-positive and VMP-positive WT cells (Fig. S2), indicating that capofungin at the inhibitory concentration enhanced PM damage and VMP. Together, the enhanced PM damage and VMP in the *TCB* mutants confirmed higher caspofungin sensitivity of the mutants than that of WT.

### Deletion of *TCB* genes has no effect on cell wall composition.

**FIG 3:**
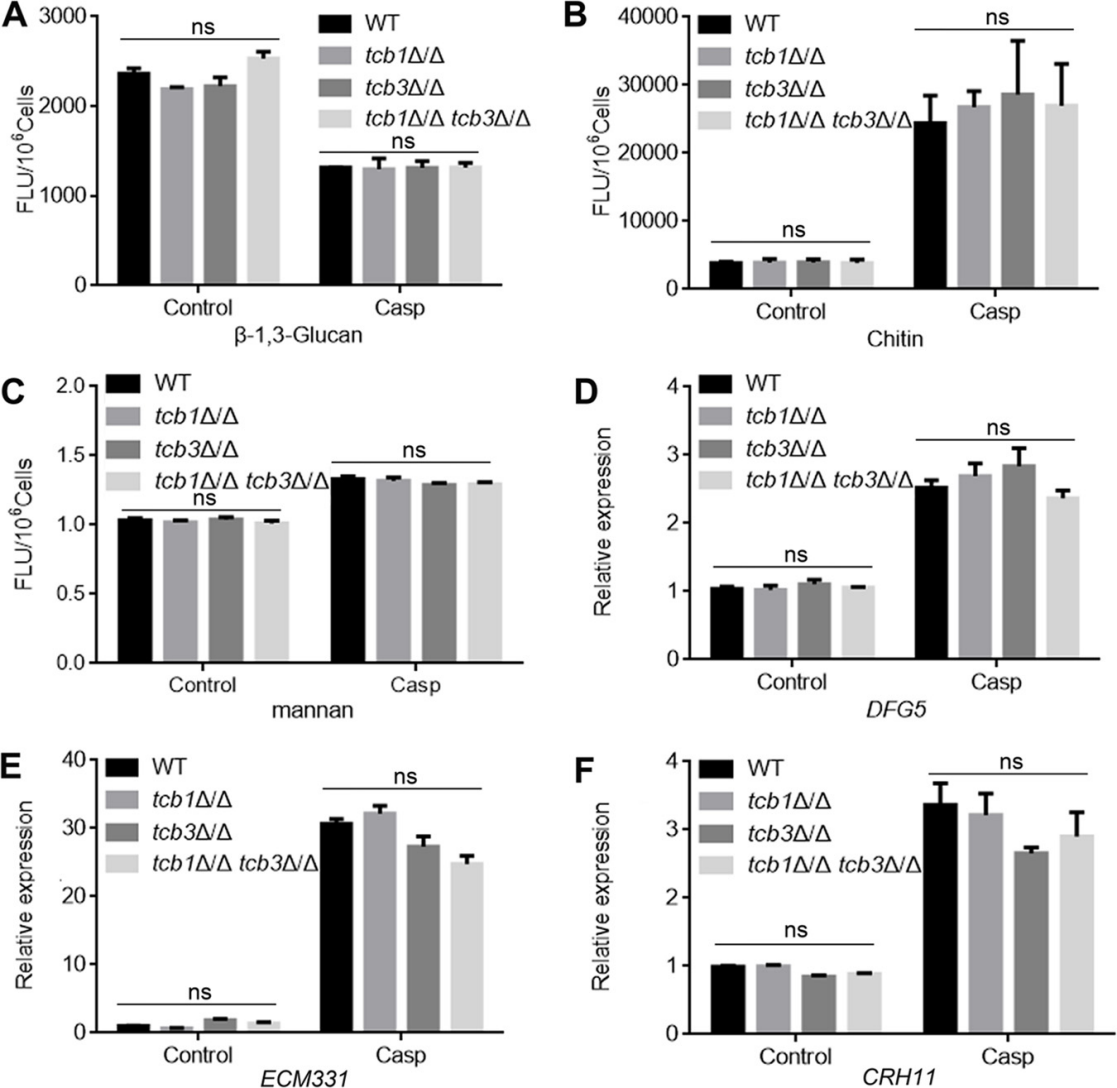
Effects of *TCB* deletion on cell wall composition (A, B, C) and expression of CWI genes (D, E, F). (A) β-1,3-glucan contents. The fungal cells were treated by 0.2 μg/mL Casp for 2 h. β-1,3-glucan contents of the treated cells were determined by Aniline Blue staining. (B) Chitin contents in the treated fungal cells revealed by CFW staining. (C) Mannan contents revealed by Alcian Blue staining. (D) Expression levels of *DFG5*. The treated cells were treated with Casp for 2 h. RNA was extracted from the cells, and cDNA was obtained by reverse transcription using Oligo (dT)-Primed RT reagent kit. The expression levels of *DFG5* were measured by RT-PCR using *ACT1* as the normalization gene. (E) Expression levels of *ECM331*. (F) Expression levels of C*RH11*. The values represent mean ± standard deviation from three biological replications. ns, no significant difference between the mutants and the WT strain, *P* < 0.05.

Cell wall composition contributes to maintenance of CWI (29). To investigate whether the caspofungin sensitivity of the mutants is attributed to altered cell wall composition, the contents of the representative cell wall components, e.g., β-1, 3-glucan, chitin, and mannan, were measured in the four strains. Interestingly, under both the control and the caspofungin-treated conditions, there was no significant difference in the cell wall β-1, 3-glucan (Fig. 3A), chitin (Fig. 3B), and mannan (Fig. 3C) between WT and the *TCB* mutants. RT-PCR assays further showed that the three mutants shared similar expression levels of the CWI genes, such as *DFG5* (Fig. 3D), *ECM331* (Fig. 3E), and *CRH11* (Fig. 3F). Therefore, the caspofungin hypersensitivity of the *TCB* mutants was not attributed to alteration of cell wall composition and expression of CWI genes.

### Deletion of *TCB* genes enhances ROS accumulation under caspofungin treatment.

**FIG 4:**
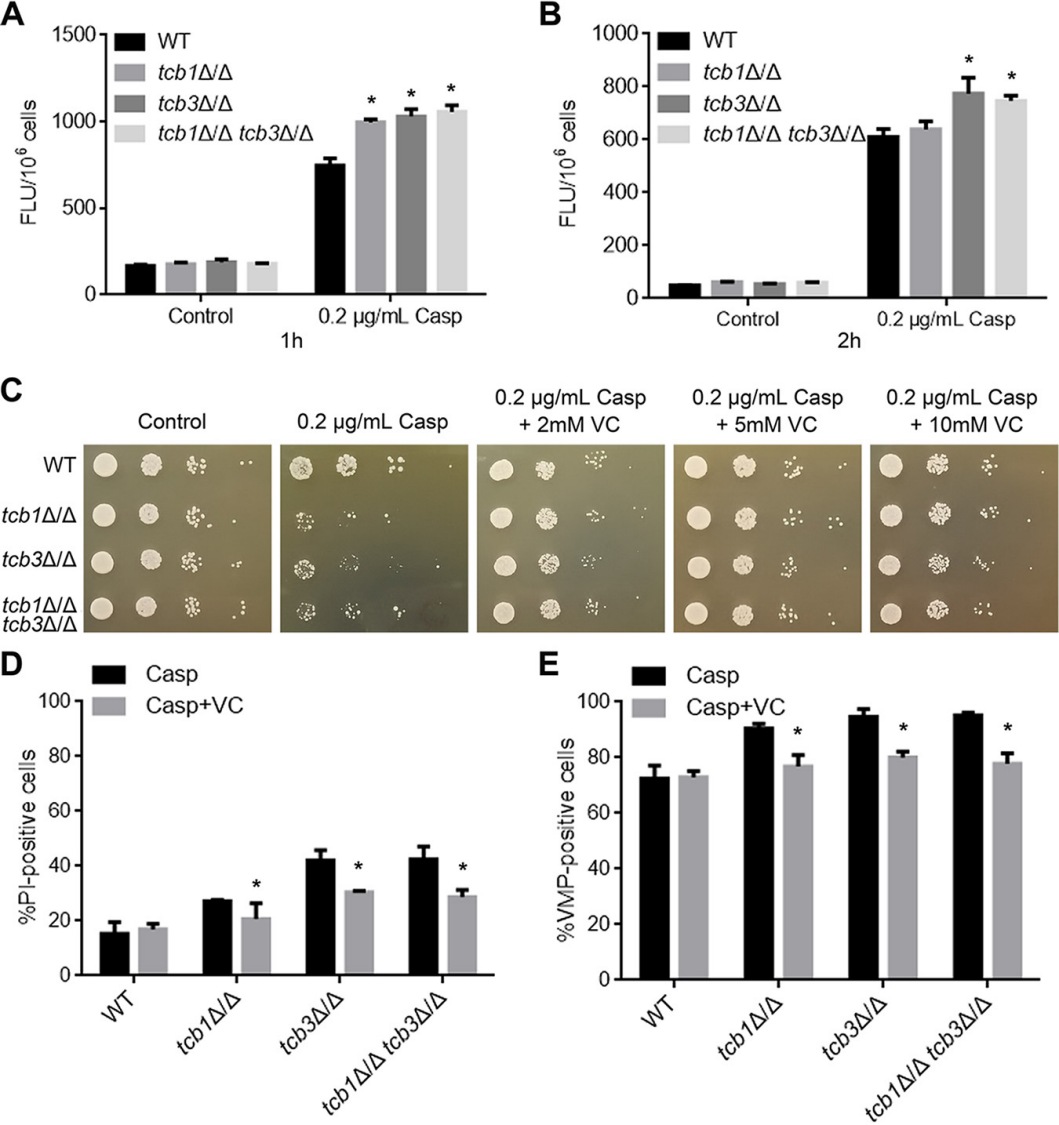
Effects of *TCB* deletion on ROS accumulation. (A) ROS levels in the cells treated by Casp for 1 h. The treated cells were stained by DCFH-DA for measurement of fluorescence intensity. (B) ROS levels in the cells treated by Casp for 2 h. (C) Effect of VC addition on growth of the strains on the plates containing 0.2 μg/mL Casp. The plates were fully cultured at 30°C for 2 days and photographed. (D) Percent of PI-positive cells after treatment of Casp alone or Casp plus VC. (E) Percent of VMP-positive cells. The values represent mean ± standard deviation from three biological replications. *, significant differences between the mutants and the WT strain, *P* < 0.05.

There is evidence that caspofungin could increase intracellular ROS levels in C. albicans and cause cell death (25). Therefore, it is speculated that the caspofungin hypersensitivity of the *TCB* deletion mutants may be associated with ROS accumulation. To confirm this, DCFH-DA staining was used to detect intracellular ROS levels. Under no caspofungin treatment, there was no significant difference in ROS levels between the *TCB* mutants and WT. However, after 1 h of caspofungin treatment, the ROS levels of the *TCB* mutants were significantly higher than that of the WT strain (Fig. 4A). After 2 h of treatment, the ROS levels of *tcb3*Δ/Δ and *tcb1*Δ/Δ *tcb3*Δ/Δ remained remarkable higher than WT (Fig. 4B). These results indicated caspofungin induced ROS accumulation in the *TCB* mutants, which may be involved in the enhanced caspofungin sensitivity.

The ROS scavenger vitamin C (VC) was then used to confirm the contribution of ROS accumulation in caspofungin sensitivity of the mutants. As expected, the addition of VC at a concentration of 2 mM, 5 mM or 10 mM rescued the growth defect of the three mutants under caspofungin treatment (Fig. 4C), indicating that VC could attenuate the inhibitory effect of caspofungin on the growth of *TCB* deletion mutants.

PM damage and VMP were further evaluated in the fungal strains under caspofungin treatment with the addition of VC. Consistent with the results of sensitivity assays, VC reduced the percentage of PI-positive cells (Fig. 4D), together with the percentage of VMP-positive cells of the three mutants (Fig. 4E). Together, these results revealed that the impaired growth of the *TCB* deletion mutants caused by caspofungin was associated with ROS accumulation in these mutants.

### Deletion of *TCB* genes increases Ero1 oxidation under caspofungin treatment.

**FIG 5:**
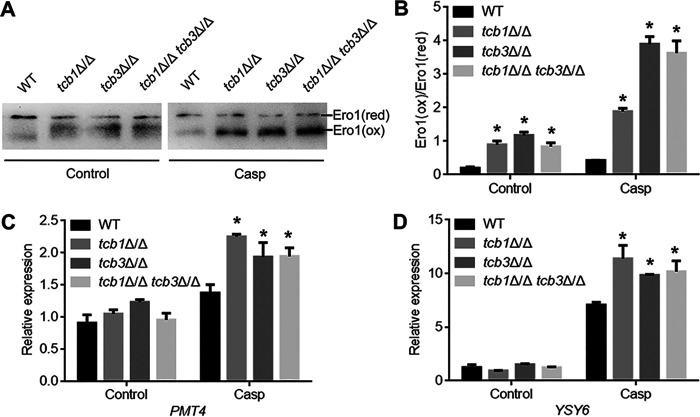
Effects of *TCB* deletion on Ero1 oxidation (A, B) and expression of the UPR genes *PMT4* and *YSY6* (C, D). (A) Western blotting images of Ero1-HA. (B) The ratio of Ero1 (ox) to Ero1 (red) revealed by Image J analysis. (C) Expression levels of *PMT4*. (D) Expression levels of *YSY6*. The values represent mean ± standard deviation from three biological replications. *, significant differences between the mutants and the WT strain, *P* < 0.05.

Ero1 is a conserved oxidoreductase in the ER that is critical for protein disulfide bond formation (30), which constitutes the main source of ER-derived ROS (31). We previously found that cell wall stress agents (e.g., CFW, caspofungin) enhanced Ero1 oxidation in the ER, leading to ROS accumulation and fungal cell death (25). To investigate whether caspofungin-induced ROS accumulation in the mutants is also associated with Ero1 oxidation, Ero1 was labeled by HA in the *TCB* mutants for Ero1 oxidation assays using the alkylation agent AMS (25). The reductive Ero1 (Ero1 [red]) containing high-level free cysteines could be alkylated by AMS, while the oxidative Ero1 (Ero1 [ox]) contains no or low-level free cysteine and could not be alkylated. Therefore, after alkylation modification, the molecular weight of Ero1 (red) is greater than Ero1 (ox) [27–29]. As revealed by Western blotting, the three *TCB* mutants had higher levels of Ero1 (ox) than WT under the control condition (Fig. 5A and B). More strikingly, under the caspofungin treatment, the mutants displayed much more severe Ero1 oxidation, while the WT strain remained quite low levels of Ero1 (ox) (Fig. 5A and B). Therefore, caspofungin severely induced Ero1 oxidation, which may contribute to ROS accumulation and caspofungin sensitivity in the mutants.

Ero1 oxidation is associated with accumulation of unfolded proteins within the ER, which further activates unfolded protein response (UPR) (32). Therefore, it is speculated that enhancement of Ero1 oxidation in the mutants may lead to ER stress and activate the UPR to relieve ER stress. We then detected the expression levels of UPR genes (i.e., *PMT4* and *YSY6*) by RT-PCR (25, 30). After caspofungin treatment, the expression levels of these two UPR genes in the *TCB* mutants were significantly higher than that in the WT strain (Fig. 5C, D), indicating that caspofungin-induced ER stress in the *TCB* mutants may be attributed to overoxidation of Ero1.

Mitochondrial dysfunction is another important cause of intracellular ROS accumulation (33, 34). Mitochondrial membrane potential (MMP) was then detected by JC-1 staining and flow cytometry to evaluate mitochondrial function. The results showed that caspofungin had no obvious impact on MMP in both WT and the *TCB* mutants (Fig. S3), indicating the normal mitochondrial function in the mutants. Therefore, Ero1 oxidation rather than mitochondrial dysfunction contributed to ROS accumulation in the *TCB* mutants under caspofungin treatment.

### Deletion of *TCB* genes impairs protease secretion and Hwp1 transport.

**FIG 6:**
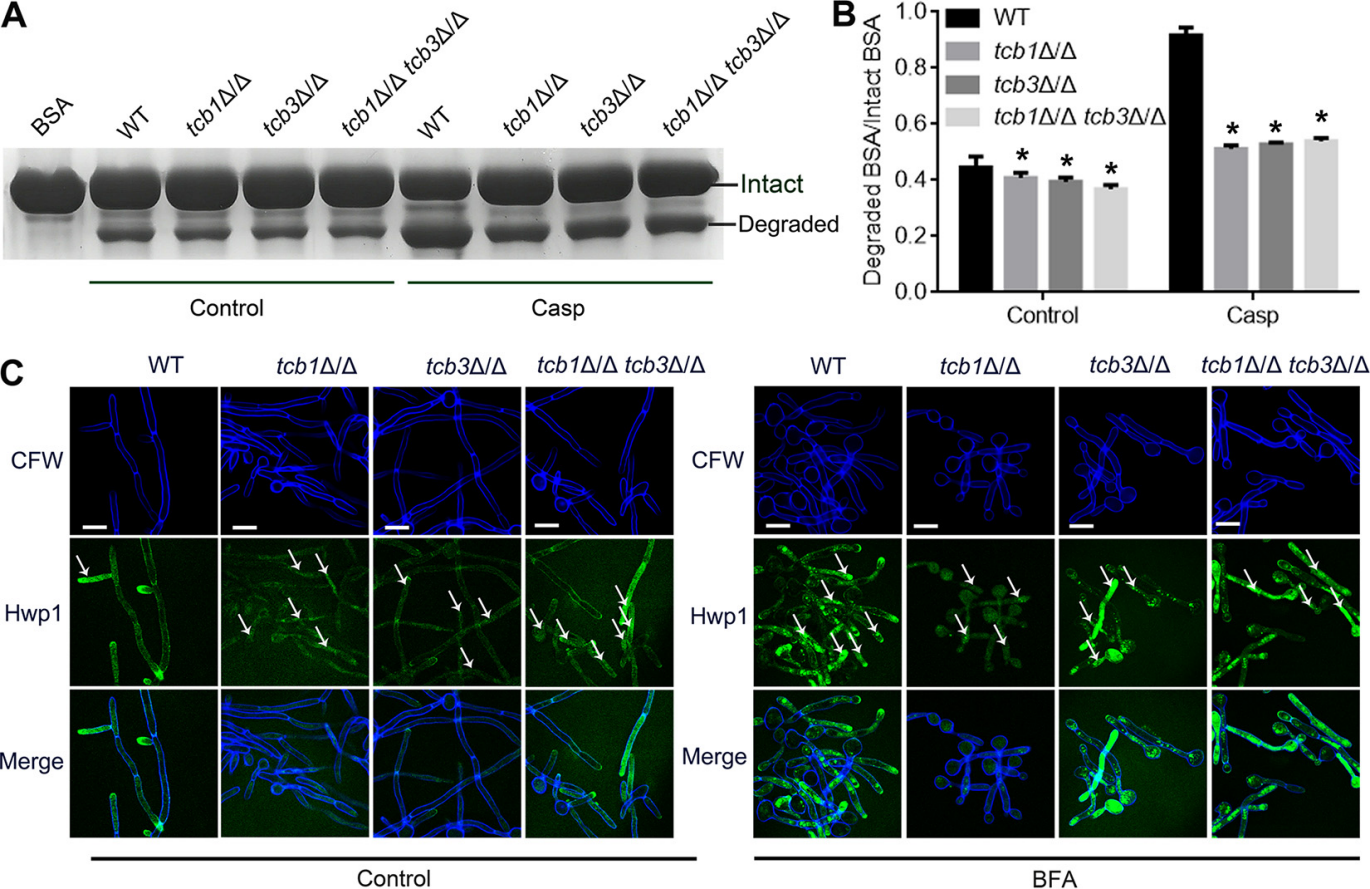
Effects of *TCB* deletion on protease secretion (A, B) and Hwp1 transport (C). (A) SDS-PAGE image of BSA after incubation with the fungal cells for 3 h. The bands were visualized by Coomassie bright blue staining. (B) The ratio of intact BSA to degraded BSA analyzed by the Image J software. (C) Confocal images of the strains, indicating distribution of Hwp1. The C. albicans cells were cultured in RPMI 1640 medium containing 250 μg/mL BFA or not for 4 h. The cells were then stained by CFW and observed with a confocal microscope. The white arrows indicate the hyphae in which Hwp1-GFP distributes in the cytosol. Scale bar = 5 μm. The values represent mean ± standard deviation from three biological replications. *, significant differences between the mutants and the WT strain, *P* < 0.05.

A portion of PM proteins, cell wall proteins, and extracellular proteins are synthesized in the ER, and then are transported to the cell surface by the conventional secretory pathway (35, 36). Since deletion of the *TCB* genes resulted in the enhancement of Ero1 oxidation for disulfide bond formation, we hypothesized that this enhancement was associated with an impairment of protein transport from the ER to the cell wall. To verify this, we detected the effect of *TCB* gene deletion on protein transport by protease secretion and Hwp1 transport assays. Under the normal condition, the *TCB* mutants had significantly lower protease secretion than WT (Fig. 6A and B). Furthermore, under the caspofungin treatment, protease secretion in each strain was significantly enhanced, indicating that caspofungin-induced cell wall stress promoted protein secretion. Compared to the WT strain, the three *TCB* mutants displayed much lower secreted protease activity (Fig. 6A and B). These results revealed that *TCB* deletion impaired protease secretion.

Hwp1 is an important adhesin in C. albicans (37), which is localized to the hyphal cell wall and could be used as an indicator of cell wall protein transport (38). We then labeled Hwp1 by GFP in the four strains to further evaluate the capacity of cell wall protein transport. It was shown that high levels of WT hyphal cells displayed Hwp1-GFP on the cell wall under the hypha-inducing condition (Fig. 6C, left). In contrast, the single mutants *tcb1*Δ/Δ and *tcb3*Δ/Δ had partial GFP fluorescence in the cytoplasm together with the cell wall, and the double mutant *tcb1*Δ/Δ *tcb3*Δ/Δ had abundant hyphal cells with Hwp1-GFP mainly distributing in the cytoplasm (Fig. 6C, left). Therefore, deletion of the *TCB* genes partially impaired cell wall protein transport.

To investigate whether the decreased Hwp1-GFP transport was attributed to an impairment of the vesicle-mediated secretion pathway, brefeldin A (BFA) was used to treat the fungal cells under the hyphal induction condition. BFA is an inhibitor of vesicle-mediated protein transport by blocking vesicle transport from the ER to the Golgi (39). As shown in Fig. 6C (right), BFA rendered Hwp1-GFP distributing both on the cell wall and in the cytoplasm in the WT strain. Thus, Hwp1-GFP transport in this control strain was only partially inhibited by BFA. This indicated that Hwp1-GFP is transported to the cell wall by two pathways, i.e., the vesicle-dependent pathway and the vesicle-independent pathway. Moreover, in the three *TCB* mutants, BFA led to almost thorough cytoplasm distribution of Hwp1-GFP, indicating cell Hwp1-GFP transport to the cell wall was almost fully impaired by BFA in the mutants. Moreover, BFA did not lead to obvious PM damage (Fig. S4), indicating that the impaired transport of Hwp1-GFP was not attributed to a leakage of the protein from lysed cells. These results indicated that tricalbins play an important role in mediating cell wall protein transport in a vesicle-independent way.

### Deletion of *TCB* genes strongly attenuates virulence of C. albicans.

**FIG 7:**
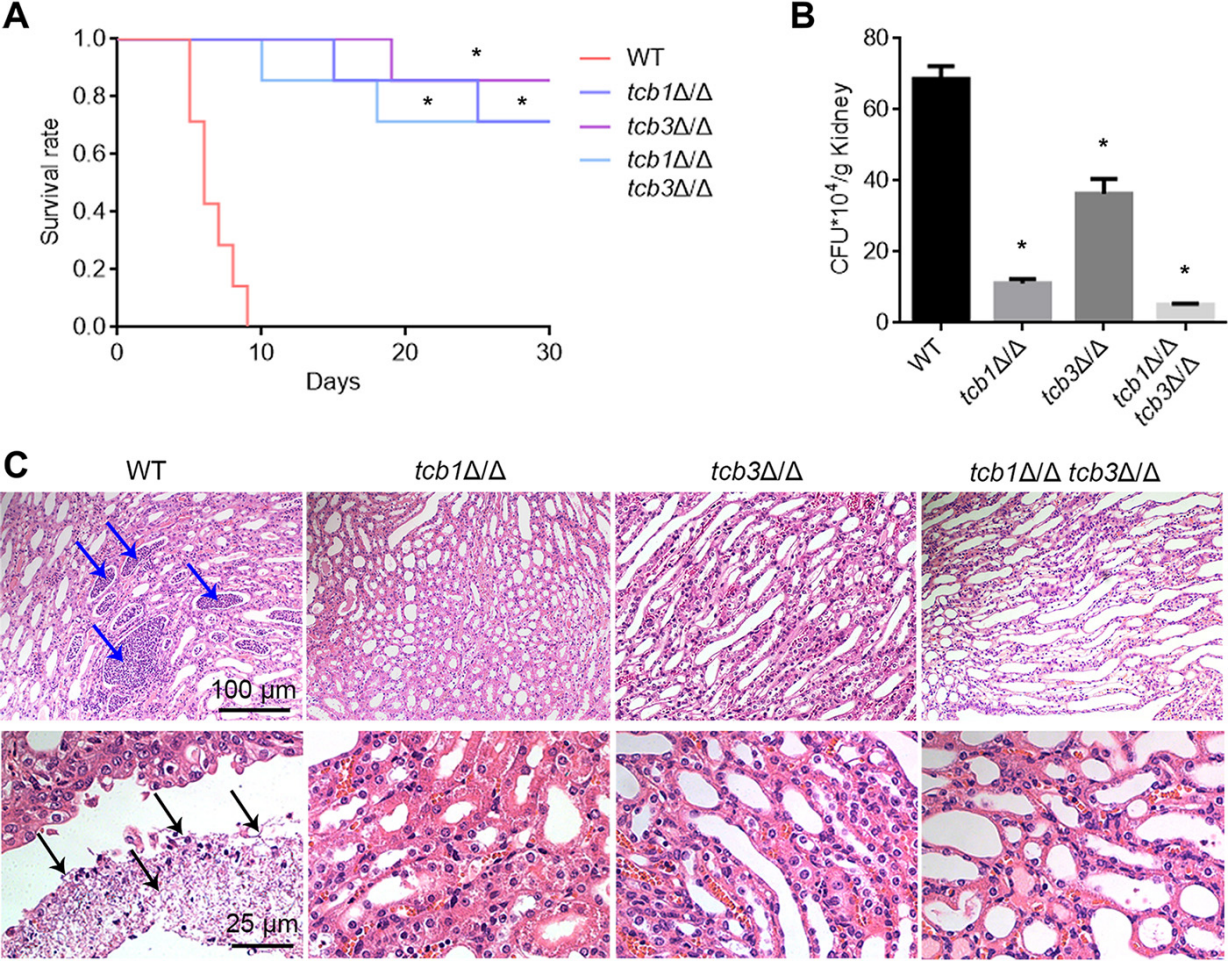
Effect of *TCB* deletion on virulence of C. albicans in the mouse systemic infection model. (A) Mice survival curves. 8 × 10^6^ C. albicans cells were injected into female ICR mice through tail veins, and survival rate of the mice were recorded every day for 30 days after injection. (B) Fungal burden in the mouse kidneys on day 5 after infection. (C) Histopathological images of mouse kidneys. The blue arrows indicate severe inflammation areas, while the black arrows indicate fungal hyphae penetrating the kidney tissues. The values represent mean ± standard deviation from three biological replications. * significant differences between the mutants and the WT strain, *P* < 0.05.

Both protease secretion and cell wall protein transport are closely related to virulence of C. albicans (40). In order to clarify the contribution of tricalbins on virulence of this pathogen, a mouse model of systemic infection was used. Since the *URA3* gene strongly affects the virulence of C. albicans, the *URA3* gene was reintroduced into the strains by pLUBP for further virulence assays (41). Mice survival curves showed that all mice injected by the WT cells were dead within 10 days, while most of the mice injected with the *TCB* mutants remained alive even on day 30 after infection (Fig. 7A). CFU assays further showed that the mice infected by the mutant cells had much lower fungal burden in the kidneys than the mice infected by the WT cells (Fig. 7B). Consistently, as revealed by histopathological observation, the kidneys infected by WT suffered from severe inflammation, and had abundant C. albicans hyphal cells penetrating the tissues. In contrast, the kidneys infected by the mutants only exhibited slight inflammation, with no fungal hypha observed (Fig. 7C, Fig. S5). These results suggested that tricalbins play an important role in the pathogenicity of C. albicans.

## DISCUSSION

The ER-PM contact is a research hot spot of cell biology. However, most of the knowledge about this structure is derived from studies of S. cerevisiae and mammalian cells (10, 12). Up to now, little is known about the components and functions of ER-PM contents in fungal pathogens. In this study, we investigated the role of the tricalbin-family ER-PM tethering proteins in formation of ER-PM contacts and cell wall stress tolerance in the fungal pathogen C. albicans. Our study revealed the significance of tricalbins in maintaining ER-PM contacts, which are involved in the survival of the pathogen under caspofungin treatment. Therefore, this study provides a novel role of ER-PM contacts in maintenance of CWI and stress response in fungal pathogens.

Caspofungin is an important and effective drug for treatment of C. albicans infections (42). As an inhibitor of β-1, 3-glucan synthetase, this drug decreases the contents of β-1, 3-glucan in the cell wall and increases the contents of chitin and mannan (23). In addition, in the face of cell wall stress caused by caspofungin, the C. albicans cells will activate the CWI pathway and upregulate the expression of CWI genes, such as *ECM331*, *CRH11*, and *DFG5* (43). We demonstrated herein that loss of tricalbins in this pathogen led to caspofungin hypersensitivity. Interestingly, further experiments showed that the caspofungin hypersensitivity of the *TCB* mutants was not caused by alteration of cell wall composition and CWI genes expression. Previous studies in our lab have found that cell wall stress may cause intracellular ROS accumulation (25). We speculated that the caspofungin hypersensitivity of the *TCB* mutants was caused by an increase of intracellular ROS levels. As expected, the mutants had a high level of ROS, and the addition of the ROS scavenger VC restored the growth of these mutants, and attenuated PM damage and VMP under caspofungin treatment. This suggests that tricalbins are involved in maintaining intracellular redox homeostasis when the fungal cells suffer from cell wall stress.

**FIG 8:**
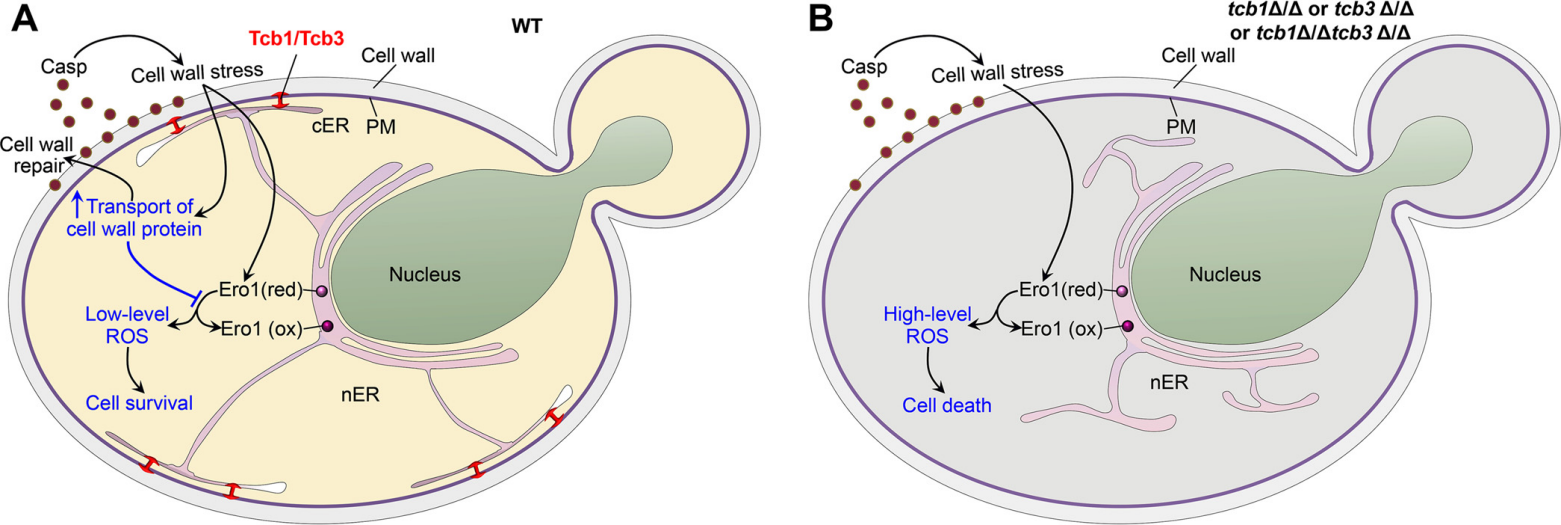
A model of tricalbin-mediated attenuation of ROS-involved caspofungin sensitivity. (A) Tricalbin-facilitated cell survival in the WT cells. The tricalbins Tcb1/Tcb3 maintain efficient transport of cell wall proteins, which alleviates Ero1 oxidation and ROS accumulation for cell survival under caspofungin treatment. (B) Cell death induced by caspofungin in the *TCB* mutants. The mutants have a defect in transport of cell wall proteins, and therefore could not alleviate Ero1 oxidation induced by caspofungin, leading to high-level ROS and consequent cell death.

In this study, the accumulation of intracellular ROS under cell wall stress in the *TCB* mutants is correlated with enhanced Ero1 oxidation, which could be induced by cell wall stress (25). On the other hand, the mutants had a defect in protease secretion and Hwp1 transport, which indicates that the cell wall protein transport was partially impaired in the mutants. Since cell wall protein transport from the ER to the cell wall is critical for cell wall repair when the cells undertake cell wall stress (44), the impairment of cell wall protein transport will no doubt deteriorate cell wall stress and further enhance Ero1 oxidation. Based on these results, we propose a model of tricalbin-mediated attenuation of ROS-involved caspofungin sensitivity (Fig. 8). In the WT cells, caspofungin-induced cell wall stress enhances Ero1 oxidation, and simultaneously promotes transport of cell wall proteins dependent on tricalbin-building ER-PM contacts. The promotion of cell wall protein transport further facilitates cell wall repair and attenuates cell wall stress, leading to alleviation of Ero1 oxidation and consequent decrease in ROS levels. Thus, the tricalbin-assisted maintenance of redox homeostasis with low-level ROS renders the WT cells surviving in caspofungin treatment (Fig. 8A). However, in the *TCB* mutants, the transport of cell wall protein is deficient and could not alleviate Ero1 oxidation under the cell wall stress. Therefore, this stress drastically promotes Ero1 oxidation and accumulation of ROS, leading to oxidative damage and consequent cell death (Fig. 8B).

In conclusion, this study reveals an unexpected role of tricalbins in cell wall stress response in the fungal pathogen C. albicans. Deletion of the *TCB* genes led to the defect in the formation of ER-PM contacts and hypersensitivity to the cell wall-perturbing drug caspofungin. This caspofungin hypersensitivity of the *TCB* mutants is attributed to Ero1 oxidation-related ROS accumulation, which is associated with the impairment of cell wall protein transport in the mutants. This study uncovers the relationship between the ER-PM contact, cell wall stress response, and maintenance of redox homeostasis in fungal cells.

## MATERIALS AND METHODS

### Strains and growth conditions.

The strains, the plasmids, and the primers used in this study are listed in Table S1, S2, S3, and S4.

All strains were constructed based on the initial C. albicans strain BWP17 (WT, *ura3*Δ/Δ, *his1*Δ/Δ, *arg4*Δ/Δ). The PCR homologous recombination method was used to knock out the two genes, i.e., *TCB1* and *TCB3* (45). To construct the single mutant *tcb1*Δ/Δ, *tcb1::ARG4* was amplified from pRS-ARG4Δ*Spe* I by PCR using primers TCB1-5DR and TCB1-3DR, and then was transformed into the BWP17 strain. The obtained heterozygous mutant *tcb1::ARG4/TCB1* was further transformed by the *tcb1::URA3-dpl200* cassette amplified from the pDDB57 plasmid, obtaining the homozygous *tcb1::ARG4/tcb1::URA3-dpl200*. The mutant was verified by the detection primers TCB1-5det and TCB1-3det. For further genetic manipulation of the homozygous mutant by using the *URA3* selection marker, the *tcb1::ARG4/tcb1::dpl200* (i.e., *tcb1*Δ/Δ) strain lossing the *URA3* marker was selected on the synthetic complete (SC) medium containing 0.1% (wt/vol) 5-FOA (80 μg/mL uridine). The *TCB3*-deleting strain *tcb1::ARG4/tcb1::dpl200* (i.e., *tcb3*Δ/Δ) was constructed in the same way.

The double deletion strain *tcb1*Δ/Δ *tcb3*Δ/Δ was obtained by using *tcb3*Δ/Δ as the starting strain, using pDDB57 as the template, and using primers (TCB1-5DR, TCB1-3DR) to amplify the *tcb1::URA3-dpl200* cassette for transformation. First, the *URA3*-depleted *tcb3*Δ/Δ mutant was transformed with the *tcb1::URA3-dpl200* cassette, generating the *TCB1* heterozygous mutant, followed by *URA3* depletion, obtaining the *URA3*-depleted *TCB1* heterozygous mutant *tcb3*Δ/Δ *TCB1::dpl200*. This strain was further transformed again with the *tcb1::URA3-dpl200* cassette, generating the *TCB1* homozygous mutant. Finally, the double deletion strain with *URA3* depletion (*tcb1*Δ/Δ *tcb3*Δ/Δ) was selected on the SC medium containing 0.1% (wt/vol) 5-FOA.

In order to obtain the localization strain WT+Tcb1-GFP, the GFP-URA3 fragment was amplified from the plasmid pGFP-URA3 by the primers TCB1-GFP-1 and TCB1-GFP-2, and then transformed into BWP17. The localization strain WT+Tcb3-GFP was constructed by the similar method. The localization strains WT+Tcb1-GFP and WT+Tcb3-GFP were further transformed with the *Nru* I-digested plasmid pDDB78-Sec61-RFP or pDDB78-PH3-RFP (46), obtaining the final localization strains WT+Sec61-RFP Tcb1-GFP, WT+Sec61-RFP Tcb3-GFP, WT+PH3-RFP Tcb1-GFP, and WT+PH3-RFP Tcb3-GFP. The localization strains WT+Sec61-GFP PH3-RFP, *tcb1*Δ/Δ+Sec61-GFP PH3-RFP, *tcb3*Δ/Δ+Sec61-GFP PH3-RFP, and *tcb1*Δ/Δ*tcb3*Δ/Δ+Sec61-GFP PH3-RFP were constructed by first transformation of the strains WT, *tcb1*Δ/Δ, *tcb3*Δ/Δ, and *tcb1*Δ/Δ*tcb3*Δ/Δ by the plasmid pAU34M-Sec61-GFP, followed by further transformation by the plasmid pDDB78-PH3-RFP.

To determine the oxidation status of Ero1, the strains was labeled with the hemagglutinin (HA) tag at the end of the *ERO1* open reading frame. YPD culture medium (yeast extract 1% [wt/vol], peptone 2% [wt/vol], glucose 2% [wt/vol], 80 μg/mL uridine) was used to culture C. albicans at a constant temperature of 30°C and 160 rpm. SC medium was prepared using yeast nitrogen without amino acids 0.67% (wt/vol), glucose 2% (wt/vol), complete amino acid mixture 0.2% (wt/vol), and uridine 80 μg/mL.

### Nucleus staining and fluorescence microscopy.

The overnight cultured C. albicans cells were added into liquid YPD medium to an optical density at 600 nm (OD_600_) of 0.1 and cultured with shaking at 30°C for 4 h. The cells were harvested and washed once with PBS (NaCl 0.8% [wt/vol], KCl 0.02% (wt/vol), Na_2_HPO_4_ 0.142% (wt/vol), KH_2_PO_4_ 0.027% (wt/vol), pH 7.4), and then treated by 1 mL 4% (wt/vol) formaldehyde for 10 min at room temperature. The fixed cells were washed once by PBS, resuspended in 1 mL PBS, and stained by 4′,6-diamidino-2-phenylindole (DAPI, 5 mg/liter, Sigma, USA) for 5 min. The cells were further washed twice with PBS and observed by a fluorescence microscope (Olympus, Japan). The quantitative data were obtained by numbering the cells displaying ER-PM contacts and the total cells in each observed field, and 20 fields were observed and numbered. The percentage of cells with ER-PM contacts were then calculated. The *t* test was applied for statistical analysis.

### Stress susceptibility test.

To test the sensitivity of C. albicans cells to the cell wall stress-inducing agent caspofungin, the overnight cultured fungal cells were suspended in YPD medium at an OD_600_ of 0.1 and then cultured with shaking at 30°C and 160 rpm for 4 h. The cells were then harvested and suspended in PBS to an OD_600_ of 0.2. The cell suspensions were dotted with 10-fold gradient dilution on the YPD solid medium containing caspofungin (dissolved in dH_2_O, final concentration 0.2 μg/mL, Sigma) or containing caspofungin plus vitamin C (2 mM, 5 mM, 10 mM). The plates were cultured at 30°C for 2 to 3 days and photographed.

### Growth curve assays.

To test the effect of caspofungin on the growth of C. albicans in liquid medium, the fungal cells were suspended in YPD medium to an OD_600_ of 0.1, and then cultured at 30°C and 160 rpm for 4 h. Caspofungin at a final concentration of 0.2 μg/mL was added into the cultures, and the cells were further cultured at 30°C and 160 rpm. The OD_600_ of C. albicans was measured every 2 h by an UV-visible spectrophotometer (Pharmacia Biotech, Swedish).

### Measurement of cell wall components.

**(i) Measurement of β-1, 3-glucan content.** The overnight cultured C. albicans cells were suspended in YPD medium to an OD_600_ of 0.1 and then were grown at 30°C and 160 rpm for 4 h. The cells were harvested and washed once with 1×TE and suspended in 500 μL 1×TE. NaOH at a final concentration of 1 M was added into the suspensions. The mixture was incubated at 80°C for 30 min. An AB mixture of 2.1 mL AB (0.03% [wt/vol] Aniline Blue, 0.18 M HCl, 0.49 M glycine, pH = 9.5) was added to the suspensions, followed by incubation at 50°C for 30 min and at room temperature for 30 min. Fluorescence intensity was measured by a fluorescence microplate reader (excitation wavelength 398 nm, emission wavelength 508 nm, PerkinElmer, USA) (47).

**(ii) Measurement of chitin content.** The cultured fungal cells were harvested and suspended in PBS. Calcofluor White (CFW) (dissolved in dH_2_O, final concentration 100 μg/mL, Sigma) was added into the suspensions, followed by incubation at 30°C for 10 min. The cells were harvested and washed twice by PBS, and fluorescence intensity was measured with a fluorescence microplate reader (excitation wavelength 325 nm, emission wavelength 435 nm). At the same time, hemocytometers were used to count the cells. The relative fluorescence intensity was calculated to reflect the chitin content of the fungal cells.

**(iii) Measurement of mannan content.** Mannan was determined by using Alcian Blue, which specifically binds phosphomannosyl residues of mannan (48). The fungal cells were harvested and washed once with 1 mL 0.02 M HCl and resuspended in 1 mL Alcian Blue solution (50 μg/mL in 0.02 M HCl). After incubation at room temperature for 10 min, the cells were pelleted. The optical density at 600 nm (OD_600_) of the supernatants was measured. The amount of Alcian Blue binding was calculated by the OD_600_ of the initial solution minus that of the supernatants, which was then divided by the number of total fungal cells to reflect the mannan contents (48).

### RNA extraction and real-time PCR (RT-PCR).

To evaluate gene expression of C. albicans, RNA was purified from frozen cells using RNA extraction kits (Promega, USA). The total cDNA was synthesized by reverse transcription using Oligo(dT) primer RT reagent kits (Promega, Madison, USA), and the levels of aimed genes were detected by SYBR green RealMastermix kits (Qiagen, China). RT-PCR was performed by using the IQ5 Multicolor Real-Time PCR Detection System (BIO-RAD, USA). The relative expression levels of the targeted CWI genes *DFG5* (encoding N-linked mannoprotein of cell wall and membrane), *ECM331* (encoding GPI-anchored protein) and *CRH11* (GPI-anchored cell wall transglycosylase), together with the UPR genes *PMT4* (encoding protein mannosyltransferase) and *YSY6* (encoding a homolog of mammalian RAMP4 protein involved in secretion), were normalized to the expression levels of *ACT1*, by using the 2^-ΔΔCT^ method. Briefly, ΔCT (mutant) was first calculated by CT (targeted gene, mutant) – CT (*ACT1*, mutant), and ΔCT (WT) was calculated by CT (targeted gene, WT) – CT (*ACT1*, WT). The relative expression of the targeted genes was then calculated by 2^-ΔΔCT^, in which ΔΔCT was calculated by ΔCT (mutant)-ΔCT (WT).

### Cellular ROS assays.

In order to determine the ROS levels of C. albicans, the overnight cultured C. albicans cells were cultured in YPD medium for 4 h, followed by 0.2 μg/mL caspofungin treatment, then harvested, suspended in PBS, and stained with 2′,7′-dichlorofluorescin diacetate (DCFH-DA, final concentration 20 μg/mL, Sigma) at 30°C for 30 min. The cells were then washed twice with PBS, resuspend with 500 μL PBS and used for determination of the fluorescence intensity by using the fluorescence microplate reader at the excitation wavelength of 488 nm and emission wavelength of 520 nm. The relative fluorescence intensity of a certain number of cells was calculated to reflect cellular ROS levels.

### Propidium iodide (PI) staining.

In order to evaluate PM damage of C. albicans, the overnight cultured C. albicans cells were cultured in YPD medium at 30°C and 160 rpm for 4 h, and then treated by caspofungin (0.2 μg/mL) or caspofungin plus vitamin C (5 mM) at 30°C and 160 rpm for 2 h. The cells were harvested and stained by PI (final concentration 1 μg/mL, Sigma) at 30°C for 10 min. The cells were harvested and washed twice with PBS for fluorescence microscopy. At least 10 fields were observed, and 100 to 200 cells in each field were counted. The percentage of PI-positive cells were then calculated.

### Vacuolar membrane permeabilization (VMP) detection.

The C. albicans cells were treated by caspofungin or caspofungin plus vitamin C as described above and harvested. The cells were then stained by 5-(6)-carboxy-2,7-dichlorofluorescein diacetate (C-DCFDA, 5 μg/mL, Sigma) at 30°C for 30 min, then washed twice by PBS, followed by fluorescence microscopy. At least 10 fields were observed. The percentage of VMP-positive cells was calculated by the number of VMP-positive cells divided by the total number of cells.

### Protease secretion assay.

Protease secretion was assayed by using bovine serum albumin (BSA) medium (complete amino acid mixture 0.2% (wt/vol), KH_2_PO_4_ 0.1% (wt/vol), MgSO_4_ 0.05% (wt/vol), glucose 2% (wt/vol), BSA 0.2% (wt/vol), pH 4.4). The freshly cultured C. albicans cells were suspended in BSA medium to an OD_600_ of 5. The cells were then cultured at 30°C and 160 rpm for 3 h. After centrifugation at 6, 000 rpm to remove fungal cells, the supernatants were used for SDS-PAGE analysis. The ratio of degraded BSA to intact BSA was analyzed by using the Image J software (V1.8.0, USA) (25).

### Detection of Ero1 oxidation.

To evaluate the state of Ero1 oxidation, The treated cells were harvested and suspended in 10% (wt/vol) trichloroacetic acid (TCA). The cells were then broken by addition of glass beads and vortexing for 30 min. After centrifugation at 12,000 rpm at 4°C for 30 min, the supernatants were obtained. Fifty μL of the supernatants were mixed with 200 μL of ice acetone, and the mixtures were placed at −20°C for 1 h. Total proteins were collected by centrifugation at 14,000 rpm for 10 min, washed with ice acetone for three times, and leaved for 10 min to allow acetone evaporation. The proteins were dissolved in SDS-AMS solution (80 mM Tris-HCl, 2% [wt/vol] SDS, 25 mM 4-acetamido-49-maleimidylstilbene-2,29-disulfonic acid [AMS], 1 mM PMSF, pH 6.8), and incubated on ice for 30 min and at 37°C for 1 h. 40 μL suspension were mixed with 10 μL 5×nonreducing SDS-PAGE buffer, and then boiled for 2 min. Western blotting was used to detect Ero1-HA using the anti-HA monoantibody (Abcam, USA). The ratio of Ero1 (ox) (oxidized Ero1, low molecular weight) to Ero1 (red) (reductive Ero1, high molecular weight) was analyzed by the Image J software (25).

### Measurement of mitochondrial membrane potential (MMP).

After treated by caspofungin (0.2 μg/mL) for 2 h, the fungal cells were stained by JC-1 (final concentration 10 μg/mL, Sigma) at 37°C for 30 min. The cells were washed twice and suspended with PBS. Red and Green fluorescence intensity of the cells was recorded by a flow cytometer (FACSCalibur, BD, USA) (25).

### Hyphal induction.

The overnight cultured C. albicans cells were suspended in liquid RPMI 1640 medium to an initial OD_600_ of 0.1, incubated at 120 rpm and 37°C for 2 h. Bafilomycin A (BFA, final concentration 250 μg/mL, Sigma) was then added into the cultures for further incubation at 120 rpm and 37°C for 4 h. The cells were harvested, washed with PBS, and stained by CFW (final concentration 5 μg/mL) for 5 min. The cells were then observed by a confocal microscope (FV1200, Olympus, Japan).

### Virulence assay.

The virulence of C. albicans was tested by a mouse systemic infection model. Initially, the C. albicans strains were transformed by the pLUBP plasmid, which contains the *URA3* gene for further recombination to the *in situ* locus of the wild-type strain genome. After pLUBP transformation, the *URA3* reconstituted strains were obtained, including WT^a^, *tcb1*Δ/Δ^a^, *tcb3*Δ/Δ^a^, and *tcb1*Δ/Δ *tcb3*Δ/Δ^a^. The four strains possessed the *in situ URA3* gene, avoiding the impact of *URA3* loss on C. albicans virulence. The overnight cultured fungal cells were suspended in 0.9% (wt/vol) NaCl to an OD_600_ of 0.8. Female 5-week-old ICR mice (15 mice in each group) were injected by 100 μL of the fungal cell suspensions via tail veins. Survival rates were recorded daily for 30 days after injection. The significance of difference between groups were analyzed by the Kaplan-Meier method. Five mice were sacrificed 5 days after injection, and the kidney fungal burdens were measured by using CFU assays. Longitudinal sections of the kidneys were also fixed with 10% (wt/vol) formalin, embedded by paraffin, sectioned, and stained with hematoxylin and eosin. The tissues were then observed by a light microscope (BX43, Olympus, Japan).

### Statistical analysis.

Each experiment was performed with three biological replicates, and three technical replicates were done for each biological replicate. The values represent the means ± standard deviation. Significant difference between the treatments was determined using the Student's *t* test (*P* < 0.05). Statistical analysis was performed using the Statistical Packages for the Social Sciences (SPSS, version 20).
