# Supplementary material for: The Tricalbin-Family Endoplasmic Reticulum-Plasma Membrane Tethering Proteins Attenuate ROS-Involved Caspofungin Sensitivity in Candida albicans
Source: Microbiol Spectr. 2022 Nov 29;10(6):e02079-22. doi: 10.1128/spectrum.02079-22 (PMC9769562; doi:10.1128/spectrum.02079-22)
Supplement: Supplemental file 2 — Fig. S1 to S5. Download spectrum.02079-22-s0002.pdf, PDF file, 0.6 MB [file spectrum.02079-22-s0002.pdf]

The tricalbin-family endoplasmic reticulum-plasma membrane tethering proteins attenuates ROS-involved caspofungin sensitivity in *Candida albicans*

Li Yang, Hangqi Zhu, Mingchun Li,* Qilin Yu*

Key Laboratory of Molecular Microbiology and Technology, Ministry of Education, Department of Microbiology, College of Life Sciences, Nankai University, Tianjin, China 300071

*Corresponding authors: Mingchun Li, nklimingchun@163.com; Qilin Yu, yuqilin@mail.nankai.edu.cn

SUPPLEMENTAL FIGURES

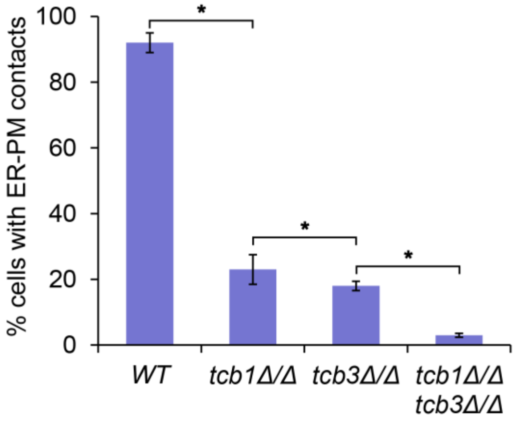

FIG S1 Statistical analysis of the cells with obvious ER-PM contacts. The asterisks (*) indicate significant difference between the groups (P < 0.05).

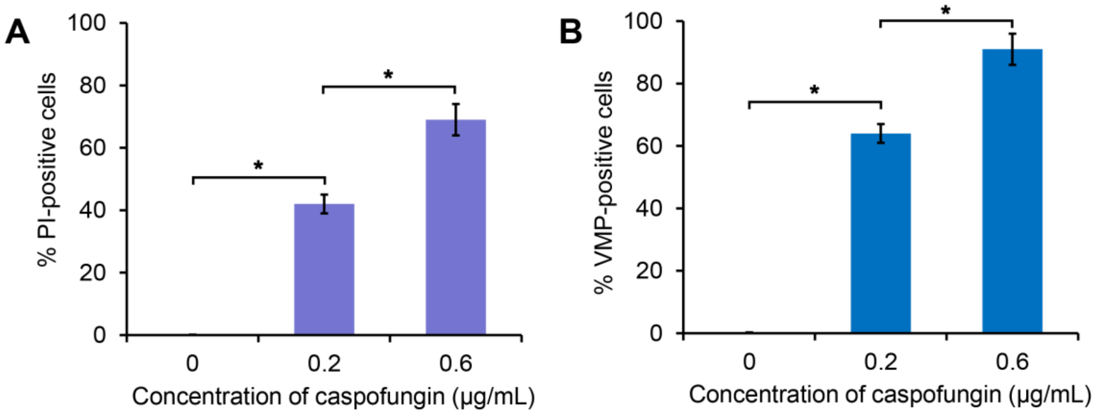

FIG S2 Impact of caspofungin on PM damage (A) and VMP (B) in WT cells.

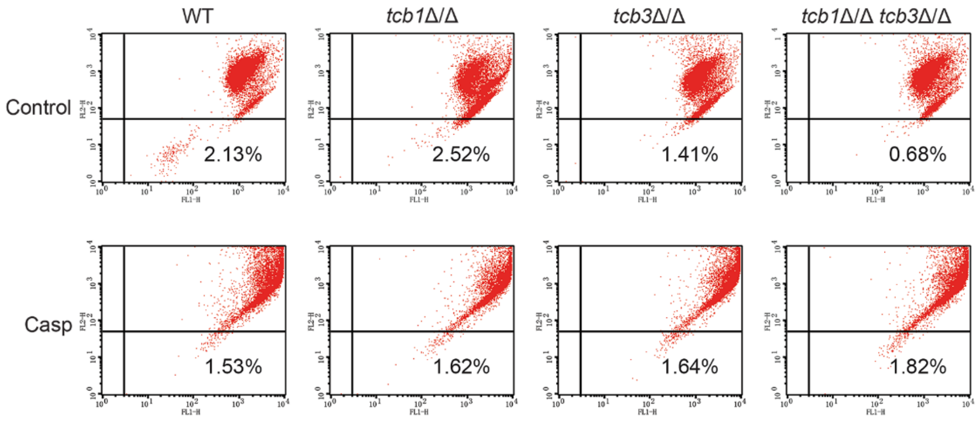

FIG S3 Effect of cell wall stress on MMP production. Cells were treated with Casp and stained with JC-1. Fluorescence density was determined by flow cytometry. The percentage of cells with decreased red fluorescence density (decreased MMP) was recorded.

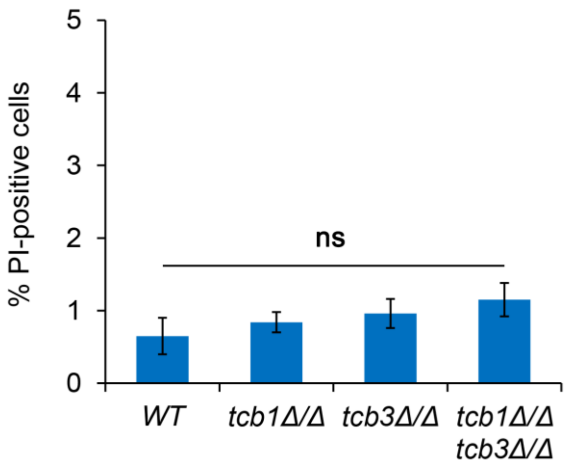

FIG S4 Impact of BFA (250 μg/mL) on PM damage in the WT and mutant cells.

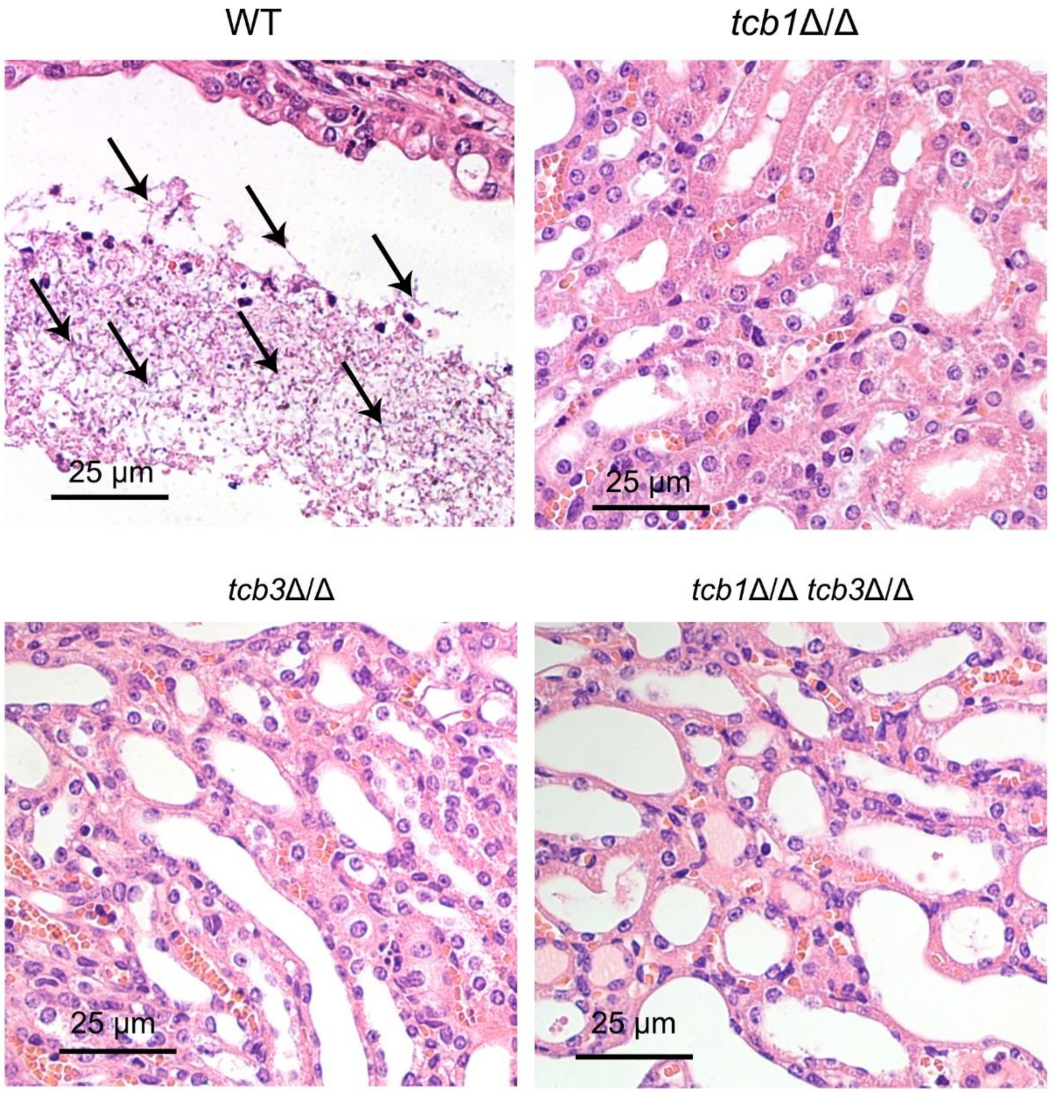

FIG S5 High-resolution histopathological images of mouse kidneys from Figure 7C. The black arrows indicate hyphal fungal cells invading the host tissues.
